# Detection of small traumatic hemorrhages using a computer-generated average human brain CT

**DOI:** 10.1117/1.JMI.5.2.024004

**Published:** 2018-05-21

**Authors:** Liza Afzali-Hashemi, Marieke Hazewinkel, Marleen C. Tjepkema-Cloostermans, Michel J. A. M. van Putten, Cornelis H. Slump

**Affiliations:** a University of Twente, MIRA Institute for Biomedical Engineering and Technical Medicine, Enschede, The Netherlands; b Medisch Spectrum Twente, Department of Neurology, Radiology and Intensive Care, Enschede, The Netherlands

**Keywords:** traumatic brain injury, computed tomography, automatic detection, image registration

## Abstract

Computed tomography is a standard diagnostic imaging technique for patients with traumatic brain injury (TBI). A limitation is the poor-to-moderate sensitivity for small traumatic hemorrhages. A pilot study using an automatic method to detect hemorrhages <10  mm in diameter in patients with TBI is presented. We have created an average image from 30 normal noncontrast CT scans that were automatically aligned using deformable image registration as implemented in Elastix software. Subsequently, the average image was aligned to the scans of TBI patients, and the hemorrhages were detected by a voxelwise subtraction of the average image from the CT scans of nine TBI patients. An experienced neuroradiologist and a radiologist in training assessed the presence of hemorrhages in the final images and determined the false positives and false negatives. The 9 CT scans contained 67 small haemorrhages, of which 97% was correctly detected by our system. The neuroradiologist detected three false positives, and the radiologist in training found two false positives. For one patient, our method showed a hemorrhagic contusion that was originally missed. Comparing individual CT scans with a computed average may assist the physicians in detecting small traumatic hemorrhages in patients with TBI.

## 1 Introduction

Traumatic brain injury (TBI) is a leading cause of morbidity and mortality with an estimated 1.6 million hospitalized and 66,000 deceased patients in Europe every year.^1^ Computed tomography (CT) is generally performed as a primary standard imaging modality for acute TBI.^2^ The advantages of CT include large-scale availability and short preparation and study time.^3^^,^^4^ CT reliably detects intracranial hemorrhages and contusions, bone pathology, cerebral edema, and incipient herniation.^4^^,^^5^ Limitations of noncontrast CT include a poor sensitivity for small hemorrhages, diffuse axonal injury (DAI), arterial dissection, and vascular damage.^6^^,^^7^ In particular, in TBI patients with secondary progression of initially undetected small cerebral hemorrhages might cause a decline in the functional status of the patient, which motivates the need of early detection of these traumatic lesions.^8^

Several approaches for automatic detection to assist in visual analysis have been proposed, ranging from machine learning on whole images^9^^,^^10^ to computer-assisted segmentations and morphological operations.^11^^,^^12^ In recent years, machine learning gained broad attention in the field of medical imaging by detecting several abnormalities. Kumar et al.^13^ introduced a robust method by expanding multivariate generalized Gaussian distribution to a reproducing kernel Hilbert space for mixture modeling. According to the authors, this statistical learning method is robust compared with the previous studies that were highly sensitive to the outliers caused by several errors, including motion and imaging artifacts. The method was examined using retinopathy and gastric and esophageal cancer datasets. After comparing the results with seven other methods, the statistical learning method showed a higher accuracy.^13^ Another study published by Schlegl et al.^14^ used generative adversarial networks to find the markers that provide information regarding the progression and treatment of a certain disease. The method was examined on 8792 two-dimensional retina images for the detection of retinal fluid and other retinal lesions. This technique was able to identify several abnormalities.

An alternative technique is comparing findings with an average head CT that was first performed by Rorden et al.^15^ A template of subjects with a mean age of 65 years was intended for the detection of brain damage in stroke patients. Gillebert et al.^16^ used this template in comparing CT scans of stroke patients and showed detection of lesion boundaries with a sensitivity of 75%.

The aim of our study was to design and realize a simple and robust automatic detection method for intracranial hemorrhages smaller than 10 mm in diameter in patients with TBI, as these are most easily missed on the CT scan images in an emergency OR situation. In some cases, secondary injury occurs if these lesions are not detected promptly. Our main goal was to develop a method with the ability to detect the presence of any type and size of intracranial hemorrhage so that the emergency room physicians can call in a neuroradiologist, a resource not likely available 24/7 in smaller regional hospitals.

## 2 Materials and Methods

### 2.1 Data Acquisition

We retrospectively collected noncontrast CT scans from the radiology database of the Medisch Spectrum Twente, The Netherlands (for details, see Appendix Table 3). As controls, we used CT scans from 30 patients (mean age 20.9, age range 18 to 24 years, 13 males) with normal findings. Two CT scans were made for reasons of persisting headache, whereas the other 28 patients were neurotrauma patients. We further collected CT images from nine TBI patients (mean age 49, age range 18 to 77 years, seven males) with hemorrhages smaller than 10 mm in diameter on the original CT. Images were obtained from TOSHIBA (Toshiba Medical Systems Corporation, Tokyo, Japan) and SIEMENS (Siemens Healthcare GmbH, Erlangen, Germany) CT scanners. CT scans with artifacts, a large amount of head asymmetry, or patients with excessive head rotation were excluded. The Medical Ethics Committee Twente waived the need for informed consent as data were obtained as part of standard care.

### 2.2 Preprocessing

Axial CT images of both the study group and control group were reconstructed to obtain a straight position of the head using IntelliSpace software (IntelliSpace Portal 7.0, Philips, Eindhoven, The Netherlands). Irrelevant slices, defined as slices above the skull and below the foramen magnum, were removed. Additionally, we reconstructed new slices with a slice thickness of 1 mm (range: 1.00 to 1.06 mm) using the thin submillimeter slices of the original scans (for details, see Appendix Table 3). Finally, the surrounding structures, such as hair, pillows, and sheets, around the brain visible on the original CT scans of the control group were removed by thresholding.

### 2.3 Registration Framework

In medical imaging, image registration is performed by spatially mapping different datasets. This procedure involves alignment of the moving image to the reference image, the fixed image.^17^ The goal of image registration is to find a coordinate transformation that ensures the spatial alignment between the fixed and moving image. The transformation, which affects the registration between the fixed and moving image, is calculated in an iterative process, in which an optimizer minimalizes the value of the cost function.^18^ In this study, registration techniques from Elastix software (Elastix Image Registration, The Netherlands) were applied.^17^^,^^19^ Using Elastix, both rigid body registration (rigid and affine) and nonrigid body registration (B-spline) were performed.^19^ The registration components are shown in Table 1.

**Table 1:** Registration steps of Elastix software with the specific components.^19^

Registration steps	Components used
Scale space to reduce data complexity	Four levels, Gaussian smoothing with standard deviation values of 4, 2, 1, and 0.5^20^^,^^21^
Image sampler	Random coordinate sampler:^18^^,^^19^^,^^22^ 3000 random coordinates
Interpolator	B-spline interpolator:^18^^,^^23^^,^^24^
	• Linear interpolation: first three levels of scale space
	• Cubic interpolation: final level of scale space
Cost function	Mutual information:^23^^,^^25^^–^^27^
	• Number of histogram bins: 32
	• Minimum required coordinate alignments to trigger the image registration: 150
Transformation	Rigid:^22^^,^^24^ translation and rotation
	Affine: translation, rotation, scaling, and shearing
	B-spline:^22^ nonrigid transformation
	• Control point spacing: 20 mm
Optimization	Adaptive stochastic gradient descent:^18^^,^^28^
	• Rigid and affine: 1500 iterations
	• B-spline transformation: 2500 iterations

### 2.4 Creating the Average Image

The CT of one of the patients in the control group was selected as the fixed image. Subsequently, all 30 CT scans (including the fixed image) were defined as moving images and were aligned to the fixed image. After image registration, all images had equal size and number of slices. Following registration, an average image from the 30 resulted images was generated. The average image was used for the detection of hemorrhages in the brains of TBI patients.

### 2.5 Detection of Small Hemorrhages

Since the skull shape, slice number, and size of the CT image of the TBI patient differed from the average image, deformation of one of the images was required. Using image registration, the average image was deformed to obtain the same features as the CT images in the TBI group. This step ensured an average image and a CT image of each TBI patient with the same dimensions but different intracranial information. The final step of this method was subtraction of the average image from the CT images of TBI patients. On the resulted images, only the tissue that is not present in the average image will remain. Since subtraction of the CT images is sensitive for the deviating densities, small hemorrhages can be detected. The resulted images were visualized using a look-up table of ITK Snap software.^29^

### 2.6 Examining the Hemorrhages

A neuroradiologist and a radiologist in training were independently asked to examine the intensity differences in the resulted images and determine the regions of hemorrhage. The clinical reports of the original CT scans were used as the gold standard. The findings of the neuroradiologist and radiologist in training were compared with the clinical notes. False positives and negatives were noted, and the sensitivity was calculated.

## 3 Results

**Fig. 1:**
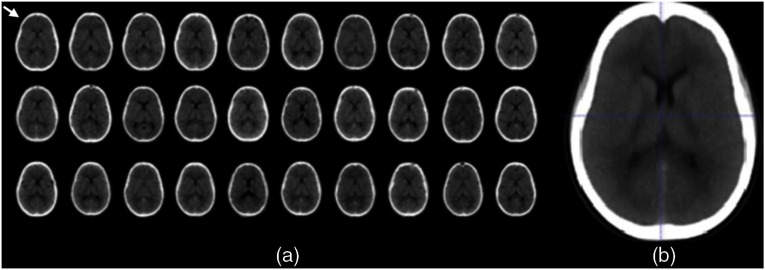
(a) The fixed image (white arrow) with 29 resulted images after image registration of the CTs of patients in the control group. All images are obtained from the same slice in the scan. The heads of the patients on the CT images have similar skull shapes with different detailed information in the brain. From these images, (b) an average image is generated.

As shown in Fig. 1(a), the skull of each patient in the control group was altered to fit the skull shape of the fixed image (white arrow), while the intracranial details differed. From these three-dimensional images, an average image was generated where each voxel in the average image represented the average of 30 voxels at that point [Fig. 1(b)].

**Fig. 2:**
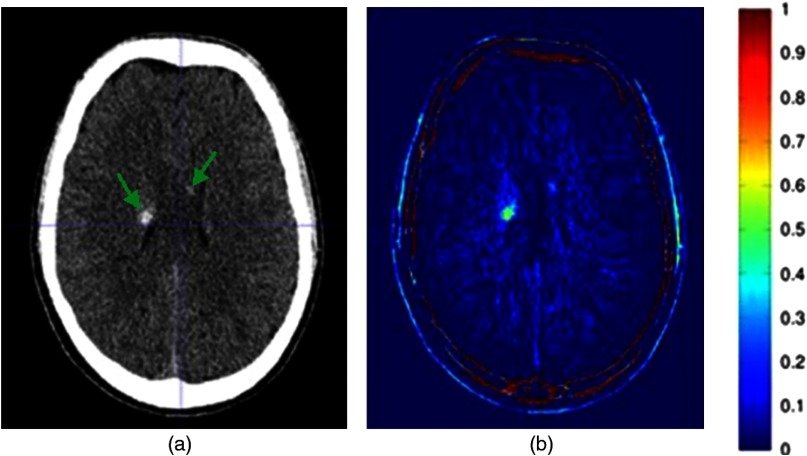
(a) A slice of the original CT image of a TBI patient with two small regions of hemorrhage (green arrows). (b) After applying the automatic detection method on the original image, the two hemorrhages were easily distinguishable from the healthy tissue. However, high intensities were also detected at the edges of the skull.

**Fig. 3:**
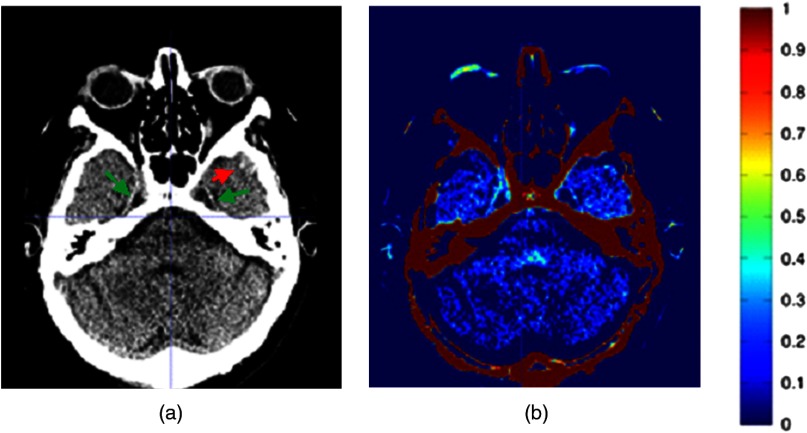
(a) A slice of the original CT image of a TBI patient with bilateral small subdural hemorrhages along Meckel’s cave (green arrows) and an originally missed cerebral hemorrhagic contusion (red arrow). (b) After applying the automatic detection method on the original image, the two subdural hemorrhages were detected, while the hemorrhagic contusion was partially detected. However, the hemorrhagic contusion was not noted in the clinical notes of the patient.

The data of the average image of patients in the control group were compared with the CT images of TBI patients, and the hemorrhages were automatically visualized. According to the clinical notes, 67 lesions were detected where the type and the region of hemorrhage differed per patient (Table 2). The neuroradiologist and the radiologist in training missed the same two hemorrhages (sensitivity of 97%) that were present in the temporal lobe near the bone. The neuroradiologist found three false positives, and the radiologist in training detected two of these false positives. Figure 2 shows one of the true positive slices of the original CT of a TBI patient and the resulting image after automatic detection. The original CT image shows two small hemorrhages at the edge of the lateral ventricles. After applying the automatic detection technique, the hemorrhages are highlighted in green/yellow. Also, the edges of the skull were visualized as a high-intensity region. Since our main goal was detection of small intracranial hemorrhages, the skull was ignored in the visual assessment. However, to maintain the anatomical orientation in the resulted image, skull stripping was not performed. For one patient, our method showed a cerebral hemorrhagic contusion that was originally missed. In the resulted images of this TBI patient, the radiologists detected a minor asymmetry in the temporal lobe, which was not noted in the clinical data of the patient. After re-examining the original CT image of the patient, a cerebral contusion in the temporal lobe was detected. Figure 3(a) shows the original CT image of the patient with bilateral small subdural hemorrhages along Meckel’s cave (green arrows) and small initially undetected hemorrhagic contusion in the left temporal area (red arrow).

**Table 2:** Type and location of hemorrhages in the brain of TBI patients.

Patients	Number of hemorrhages	Type and location of the hemorrhages
Patient 1	12	Subarachnoid: left temporal
		Cerebral contusions: left parietal and bilateral frontal and temporal
		DAI: bilateral randomly present
Patient 2	2	Cerebral contusions: left temporal
Patient 3	11	DAI: corpus callosum
		Subarachnoid: right temporal
		Intraventricular: bilateral
Patient 4	16	DAI: left parietal, temporal, and basal ganglia
		Subarachnoid: bilateral frontal
		Cerebral contusions: left temporal
Patient 5	3	DAI: near the right ventricle
		Subarachnoid: right temporal
Patient 6	14	Cerebral contusions: bilateral frontal
		DAI: bilateral randomly present
		Subdural: left frontoparietal
Patient 7	2	Subdural: bilateral temporal
Patient 8	1	Subarachnoid: right frontotemporal
Patient 9	6	Cerebral contusions: left parietal
		Subarachnoid: bilateral occipital
		DAI: bilateral randomly present

The final images of TBI patients also showed high-intensity regions along the cerebellar tentorium, pons, and around the lateral ventricles. Furthermore, the radiologists had no difficulties in discerning dense vessels and calcifications from hemorrhages in the obtained images.

## 4 Discussion

In this pilot study, we mimicked the visual analysis of the neuroradiologists with an automatic detection method by easily identifying small cerebral hemorrhages in TBI patients using a computer-generated “average brain” from 30 control patients. From 67 lesions, the automatic detection method missed only two lesions that were present in the temporal fossa near the bone. The middle temporal fossa is highly variable in shape and size per individual. These variations were observed on the original CT scans of 30 patients in the control group. Since only 30 young patients were included in the control group, the average image will be affected by these individual differences. The shape of the fossa media is partially race dependent; therefore, development of several templates based on race may solve a part of the problem.

The automatic method detected a hemorrhage that was initially missed by a resident. After detecting the hemorrhage by our automatic method, two neuroradiologists and a radiologist in training critically re-examined the original CT image. The three physicians were able to confirm the presence of hemorrhage on the original CT image.

In most hospitals, the examination of the (neuro)radiologist and the resulting clinical notes are used as a gold standard for TBI patients. Since autopsy is usually not an option and our work is a retrospective pilot study, we used the visual analysis of our neuroradiologists as the gold standard.

Al-Ayyoub et al.^9^ examined CT images of 74 patients classified into normal, epidural, subdural, and intraparenchymal hemorrhages. They applied several techniques, such as image preprocessing, segmentation, region growing, feature extraction, and classification, resulting in an accuracy of 100% for the detection of hemorrhages. Shahangian and Pourghassem^30^ performed automatic brain hemorrhage segmentation and classification algorithm based on weighted grayscale histogram feature in a hierarchical classification structure. Detection accuracies of epidural, subdural, and intracerebral hemorrhages were 96.15%, 94.87%, and 95.96%, respectively. Liu et al.^10^ used machine learning for the detection of cerebral hemorrhages in neurotrauma patients with a detection accuracy of 80%. The authors included mostly large hemorrhages that were not easily overlooked on the original CT images. In contrast to these studies, we focused on hemorrhages smaller than 10 mm in diameter. Comparing our pilot results to the results of these studies, our method generally falls in the same accuracy range. However, it is noteworthy that we have only included 9 CT scans in the TBI group and 30 CT scans in the control group. Additionally, our method did not require any segmentation of the skull, ventricles, or cerebrospinal fluid (CSF).

The original CT images of the brain are often initially read by a radiology resident or sometimes by a nonradiologist. Our automatic method may assist the radiology resident and other physicians in the detection of small traumatic hemorrhagic lesions. Since our method visualizes normal tissue with a blue color and other findings with other colors, the physicians may quickly know where to focus on the image. Automatic detection can save time by directly distinguishing lesions from healthy brain areas. Moreover, it may reduce the interobserver variation between radiologists.

In this study, we compared the CT scans of a relative young control group with TBI patients of different age groups. Since aging changes the brain, sometimes reducing it in size,^31^ it is more precise to compare patients with age-matched control groups. This may reduce misdetection and may cause less deformation of the average image to correctly align to the TBI images.

To initiate the image registration, a reference image was required to align the images. In this work, a CT image of a patient in the control group was selected as the fixed image. Since there was one fixed image and 30 moving images, the fixed image had a higher influence on the resulted images. An approach for eliminating the bias is a groupwise image registration where all images are aligned to a common space. Applying a groupwise registration, the information of all images will have an equal influence on the average image.^32^^–^^34^ Recently, Elastix introduced the method of Huizinga et al.^32^ in its database for groupwise registration of MRI images. To our knowledge, using Elastix software, this approach has not been applied on CT images.

Several registration steps were applied to obtain a good trade-off between quality and speed. The goal was to create an average brain CT from several CT images to include the normal variability in brain tissue. After applying rigid and affine transformation, the details of the brain tissue in the resulted images looked nearly the same as the fixed image. Since the images were obtained from different patients, rigid transformation was not able to correctly align the images and to include the details of the moving images in the results. To perform the registration more precisely, nonrigid B-spline transformation was applied. The B-spline method uses a grid with automatically placed control points on the vertices of the grid squares that are overlaid on the image. The spacing of the control points determines the deformation. Large spacing describes a global deformation, whereas small spacing defines a local deformation. To match the small structures in the images, different spacing values were tested. As larger values of 30 and 40 mm resulted in a mismatched registration image, a spacing of 20 mm between two control points was preferred for the CT registration. The results of nonrigid transformation showed a good brain overlap that was confirmed by visual inspection. Using nonrigid transformation, the tissue density may change locally, especially in the transition regions from white to gray matter, CSF, and parenchyma. However, these density differences are still lower than the density of a hemorrhage. The densities of CSF and brain tissue are between 3 and 40 HU, and the density of blood is generally higher than 60 HU.^35^^,^^36^ The automatic method is developed for detecting intracranial hemorrhages and will not be affected by other focal lesions, such as white matter lesions.

The resulted images of TBI patients showed higher intensity regions around the lateral ventricles. The reason for these high-intensity regions may be the large differences in contrast between the ventricles and the brain parenchyma. Since the shape and position of the ventricles differ per individual, voxel resampling to another brain may cause this misdetection. Furthermore, some TBI patients develop a midline shift that causes a displacement of the ventricles. This displacement can have an influence during the comparison of TBI scans to the control group and may cause the false positives in the regions around the ventricles. The neuroradiologist and radiologist in training did not identify the high-intensity regions around the ventricles as pathologic. It is, however, possible that other physicians may interpret these areas as false positives. A possible solution for this problem could be the segmentation of the ventricles before image registration.

Similar problematic areas were regions around the pons and/or cerebellar tentorium. A reason for these areas being highlighted could be artifacts on the original CT images that are difficult to distinguish from hemorrhage. Other reasons for the high intensities in the cerebellum could be subarachnoid blood or a consequence of our method. Subarachnoid blood is usually quickly diluted by the CSF, so it may not be present on a follow-up study. For these types of findings, prospective research with a follow-up MRI could provide additional information. Using this information, we can examine if these high-intensity regions are caused by our method or by other factors.

## 5 Conclusion

We have introduced an automatic detection method for the detection of small traumatic brain hemorrhages in TBI patients using a computer-generated average CT. Our automatic detection method showed encouraging pilot results and a good correlation with the visual analysis of the neuroradiologists. The automatic comparison of individual CT scans with the computed average may assist the physicians in early detection of small hemorrhages.

## Appendix

The patients obtained a CT scan with varied settings in some cases. A overview of these settings are listed in Table 3. Since the slice thickness differed in some scans, the images were reconstructed to equalise the thicknesses.

**Table 3:** Size and setting information of the CT images of both control and TBI group.

CT features	Control group	TBI group
Amount of slices	133 to 171	138 to 154
Peak kilovoltage (kVp)	120 kV	120 kV
X-ray tube current	124 to 452 mA	187 to 518 mA
Exposure	124 to 410 mAs	187 to 470 mAs
Pitch	0.55 to 0.66	0.55 to 0.66
Total collimation width	6 to 38.4 mm	6 to 16 mm
Pixel spacing	[0.3899;0.3899] to [0.4814;0.4814]	[0.4187;0.4187] to [0.5859;0.5859]
Matrix size	[512 512]	[512 512]
Slice thicknesses before reconstruction	0.5 to 1 mm	0.5 to 1 mm
Slice thicknesses after reconstruction	1 to 1.03 mm	1 to 1.06 mm

## References

[1] Willemse-van Son A. H. P. et al., “Prognostic factors of long-term functioning and productivity after traumatic brain injury: a systematic review of prospective cohort studies,” Clin. Rehabil. 21(11), 1024–1037 (2007).https://doi.org/10.1177/0269215507077603 17984154 10.1177/0269215507077603

[2] Metting Z. et al., “Perfusion computed tomography in the acute phase of mild head injury: regional dysfunction and prognostic value,” Ann. Neurol. 66(6), 809–816 (2009).https://doi.org/10.1002/ana.21785 20035508 10.1002/ana.21785

[3] Glauser J., “Head injury: which patients need imaging? Which test is best?” Cleveland Clin. J. Med. 71(4), 353–357 (2004).https://doi.org/10.3949/ccjm.71.4.353 10.3949/ccjm.71.4.353 15117178

[4] Amyot F. et al., “A review of the effectiveness of neuroimaging modalities for the detection of traumatic brain injury,” J. Neurotrauma 32(22), 1693–1721 (2015). JNEUE4 0897-7151 https://doi.org/10.1089/neu.2013.3306 26176603 10.1089/neu.2013.3306 PMC4651019

[5] Coles J. P., “Imaging after brain injury,” Br. J. Anaesth. 99(1), 49–60 (2007). BJANAD 0007-0912 https://doi.org/10.1093/bja/aem141 17573394 10.1093/bja/aem141

[6] Fred H. L., “Drawbacks and limitations of computed tomography: views from a medical educator,” Tex. Heart Inst. J. 31(4), 345–348 (2004). THIJDO 0730-2347 15745283 PMC548232

[7] Kim J. J. Gean A. D., “Imaging for the diagnosis and management of traumatic brain injury,” Neurotherapeutics 8(1), 39–53 (2011).https://doi.org/10.1007/s13311-010-0003-3 21274684 10.1007/s13311-010-0003-3 PMC3026928

[8] Doddamani R. S. et al., “Role of repeat CT scans in the management of traumatic brain injury,” Indian J. Neurotrauma 9(1), 33–39 (2012).https://doi.org/10.1016/j.ijnt.2012.04.007

[9] Al-Ayyoub M. et al., “Automatic detection and classification of brain hemorrhages,” WSEAS Trans. Comput. 12(10), 395–405 (2013).

[10] Liu R. et al., “Hemorrhage slices detection in brain CT images,” in 19th Int. Conf. on Pattern Recognition, 2008 (ICPR 2008), pp. 1–4 (2008).

[11] Chan T., “Computer aided detection of small acute intracranial hemorrhage on computer tomography of brain,” Comput. Med. Imaging Graphics 31(4–5), 285–298 (2007).https://doi.org/10.1016/j.compmedimag.2007.02.010 10.1016/j.compmedimag.2007.02.010 17376649

[12] Matesin M. Loncaric S. Petravic D., “A rule-based approach to stroke lesion analysis from CT brain images,” in Proc. 2nd Int. Symp. on Image Signal Processing and Analysis 2001 (ISPA 2001), pp. 219–223, IEEE (2001).https://doi.org/10.1109/ISPA.2001.938631

[13] Kumar N. et al., “Kernel generalized-Gaussian mixture model for robust abnormality detection,” in Int. Conf. on Medical Image Computing and Computer-Assisted Intervention (MICCAI 2017), Vol. 10434, pp. 21–29 (2017).

[14] Schlegl T. et al., “Unsupervised anomaly detection with generative adversarial networks to guide marker discovery,” Lect. Notes Comput. Sci. 10265, 146–147 (2017). LNCSD9 0302-9743 https://doi.org/10.1007/978-3-319-59050-9_12

[15] Rorden C. et al., “Age-specific CT and MRI templates for spatial normalization,” NeuroImage 61(4), 957–965 (2012).https://doi.org/10.1016/j.neuroimage.2012.03.020 22440645 10.1016/j.neuroimage.2012.03.020 PMC3376197

[16] Gillebert C. R. Humphreys G. W. Mantini D., “Automated delineation of stroke lesions using brain CT images,” Neuroimage Clin. 4, 540–548 (2014).https://doi.org/10.1016/j.nicl.2014.03.009 24818079 10.1016/j.nicl.2014.03.009 PMC3984449

[17] Shamonin D., “Fast parallel image registration on CPU and GPU for diagnostic classification of Alzheimer’s disease,” Front. Neuroinform. 7, 1–15 (2013).https://doi.org/10.3389/fninf.2013.00050 24474917 10.3389/fninf.2013.00050 PMC3893567

[18] Klein S. Staring M. Pluim J. P. W., “Evaluation of optimization methods for nonrigid medical image registration using mutual information and B-splines,” IEEE Trans. Image Process. 16(12), 2879–2890 (2007). IIPRE4 1057-7149 https://doi.org/10.1109/TIP.2007.909412 18092588 10.1109/tip.2007.909412

[19] Klein S. et al., “elastix: a toolbox for intensity-based medical image registration,” IEEE Trans. Med. Imaging 29(1), 196–205 (2010). ITMID4 0278-0062 https://doi.org/10.1109/TMI.2009.2035616 19923044 10.1109/TMI.2009.2035616

[20] Lester H. Arridge S. R., “A survey of hierarchical non-linear medical image registration,” Pattern Recognit. 32(1), 129–149 (1999).https://doi.org/10.1016/S0031-3203(98)00095-8

[21] Rey-Otero I. Delbracio M., “Computing an exact Gaussian scale-space,” Image Process. Line 6, 8–26 (2016).https://doi.org/10.5201/ipol.2016.117

[22] Klein S. Staring M., “elastix: the manual,” 2015 http://elastix.isi.uu.nl/download/elastix_manual_v4.8.pdf (20 7 2016).

[23] Unser M., “Splines: a perfect fit for signal and image processing,” IEEE Signal Process. Mag. 16(6), 22–38 (1999). ISPRE6 1053-5888 https://doi.org/10.1109/79.799930

[24] Ashburner J. Friston K. J., “Spatial transformation of images,” Hum. Brain Funct. 43–58 (1997).

[25] Thévenaz P. Unser M. Thevenaz P., “Optimization of mutual information for multiresolution image registration,” IEEE Trans. Image Process. 9(12), 2083–2099 (2000). IIPRE4 1057-7149 https://doi.org/10.1109/83.887976 18262946 10.1109/83.887976

[r26] Pluim J. P. W. Maintz J. B. A. Viergever M. A., “Mutual information based registration of medical images: a survey,” IEEE Trans. Med. Imaging 28, 986–1004 (2003). ITMID4 0278-0062 https://doi.org/10.1109/TMI.2003.815867 10.1109/TMI.2003.815867 12906253

[27] Maes F. et al., “Multimodality image registration by maximization of mutual information,” IEEE Trans. Med. Imaging 16(2), 187–198 (1997). ITMID4 0278-0062 https://doi.org/10.1109/42.563664 9101328 10.1109/42.563664

[28] Klein S. et al., “Adaptive stochastic gradient descent optimisation for image registration,” Int. J. Comput. Vision 81(3), 227–239 (2009). IJCVEQ 0920-5691 https://doi.org/10.1007/s11263-008-0168-y

[29] Yushkevich P. A. et al., “User-guided 3D active contour segmentation of anatomical structures: significantly improved efficiency and reliability,” NeuroImage 31(3), 1116–1128 (2006).https://doi.org/10.1016/j.neuroimage.2006.01.015 16545965 10.1016/j.neuroimage.2006.01.015

[30] Shahangian B. Pourghassem H., “Automatic brain hemorrhage segmentation and classification algorithm based on weighted grayscale histogram feature in a hierarchical classification structure,” Biocybern. Biomed. Eng. 36(1), 217–232 (2016).https://doi.org/10.1016/j.bbe.2015.12.001

[31] Peters R., “Ageing and the brain,” Postgrad. Med. J. 82, 84–88 (2006).https://doi.org/10.1002/path.2089 16461469 10.1136/pgmj.2005.036665 PMC2596698

[32] Huizinga W. et al., “PCA-based groupwise image registration for quantitative MRI,” Med. Image Anal. 29, 65–78 (2016).https://doi.org/10.1016/j.media.2015.12.004 26802910 10.1016/j.media.2015.12.004

[r33] Guyader J. M. et al., “Total correlation-based groupwise image registration for quantitative MRI,” in Proc. IEEE Conf. Computer Vision Pattern Recognition Workshop, pp. 626–633 (2016).https://doi.org/10.1109/CVPRW.2016.84

[34] Geng X. et al., “Unbiased group-wise image registration: applications in brain fiber tract atlas construction and functional connectivity analysis,” J. Med. Syst. 35(5), 921–928 (2011). JMSYDA 0148-5598 https://doi.org/10.1007/s10916-010-9509-9 20703687 10.1007/s10916-010-9509-9 PMC2987551

[35] Claussen C. et al., Computed Tomography and Magnetic Resonance Tomography of Intracranial Tumors: A Clinical Perspective, Kazner E. et al., Eds., 2nd ed., Springer-Verlag, Berlin, Heidelberg (1989).

[36] Cala L. A. et al., “Brain density and cerebrospinal fluid space size: CT of normal volunteers,” Am. J. Neuroradiol. 2(1), 41–47 (1981). 6784549 PMC8331815

